# Quantifying steric hindrance and topological obstruction to protein structure superposition

**DOI:** 10.1186/s13015-020-00180-3

**Published:** 2021-02-27

**Authors:** Peter Røgen

**Affiliations:** Department of Applied Mathematics and Computer Science, Technical University of Denmark, Asmussens Allé, Building 322, Kongens Lyngby, Denmark

**Keywords:** Protein topology, Structural classification of proteins, Self-avoiding morphs, Primary 92C40, Secondary 92E10, 57K10

## Abstract

**Background:**

In computational structural biology, structure comparison is fundamental for our understanding of proteins. Structure comparison is, e.g., algorithmically the starting point for computational studies of structural evolution and it guides our efforts to predict protein structures from their amino acid sequences. Most methods for structural alignment of protein structures optimize the distances between aligned and superimposed residue pairs, i.e., the distances traveled by the aligned and superimposed residues during linear interpolation. Considering such a linear interpolation, these methods do not differentiate if there is room for the interpolation, if it causes steric clashes, or more severely, if it changes the topology of the compared protein backbone curves.

**Results:**

To distinguish such cases, we analyze the linear interpolation between two aligned and superimposed backbones. We quantify the amount of steric clashes and find all self-intersections in a linear backbone interpolation. To determine if the self-intersections alter the protein’s backbone curve significantly or not, we present a path-finding algorithm that checks if there exists a self-avoiding path in a neighborhood of the linear interpolation. A new path is constructed by altering the linear interpolation using a novel interpretation of Reidemeister moves from knot theory working on three-dimensional curves rather than on knot diagrams. Either the algorithm finds a self-avoiding path or it returns a smallest set of essential self-intersections. Each of these indicates a significant difference between the folds of the aligned protein structures. As expected, we find at least one essential self-intersection separating most unknotted structures from a knotted structure, and we find even larger motions in proteins connected by obstruction free linear interpolations. We also find examples of homologous proteins that are differently threaded, and we find many distinct folds connected by longer but simple deformations. TM-align is one of the most restrictive alignment programs. With standard parameters, it only aligns residues superimposed within 5 Ångström distance. We find 42165 topological obstructions between aligned parts in 142068 TM-alignments. Thus, this restrictive alignment procedure still allows topological dissimilarity of the aligned parts.

**Conclusions:**

Based on the data we conclude that our program ProteinAlignmentObstruction provides significant additional information to alignment scores based solely on distances between aligned and superimposed residue pairs.

## Background

Proteins are essential cellular tools and as macroscopic tools, some shapes are preferential for fulfilling specific functions. Protein shapes are known to be more preserved than their sequences of amino acids: structural comparison of proteins is therefore an important and active area of research with new structural alignment methods reported to double every five years for three decades [1]. Aside from the rapid growth of the number of known protein structures, protein structure comparison is challenging for several reasons. One is that even for a fixed sequence of amino acids, some proteins are highly flexible [2]. Another is that mutations cause plastic deformation that comparison methods should also take into account [1]. On the other hand, proteins reuse the same types of folds, but with the growing number of known protein structures, the global view is changing from discrete folds into considering larger parts of fold space as a continuum [3].

In protein structure comparison either the shapes of proteins’ solvent accessible surfaces [4, 5] or their folded backbones are compared. Surface comparison mainly address how a protein is seen from and possibly interacts with its exterior. Chain or curve comparison mainly address how a protein is folded upon itself and is typically used when searching structural evolutionary evidence [6] and when judging efforts to predict protein structures [7]. One exception to this general trend is that surfaces of protein models generally can be discriminated from surfaces of native proteins [8]. It is not hard to imagine two long protein chains sharing a surface but being folded differently inside the surface. Here, we therefore only consider structural comparison based on curve comparison.

Most methods for pairwise curve comparison of protein structures combine methods for searching alignment and optimal superposition of subsets of two proteins with a score function assessing the quality of this alignment. See [1] for an overview of the most used methods. All these score functions are based on distances, and it is our hypothesis that in practice they are blind to topological changes even in the aligned parts as we now explain. Each score function is based on the distances that aligned residues are moved when the structures are in optimal superposition. But its not taken into account if the motion may be performed by a continuous deformation or if it will lead to self-intersections of the protein that may change its fold or even tie a knot in it. Mathematically that is, the score functions of most alignment and superposition methods report similarity or distances in the space of immersions of proteins. Theoretically, we will argue, a pair of topologically distinct embeddings of a protein may have a similarity score typically found between highly similar protein structures. This is apparently also found in practice as [9] states *“These classic structure comparison metrics”* (Root Mean Square Deviation (RMSD) [10], Global Distance Test-Total Score (GDT-TS) [7], and TM-score [11]) *“need to be supplemented by more sophisticated measures, which quantify topographical differences in chain progression in 3D space”*.

Superposition free alignment methods based on changes in internal distances such as DALI [12], FlexE [13] and lDDT [14] also do not check for topological changes. Mathematically they cannot tell a structure from its mirror image. More interestingly, local chirality changes can occur under minor changes to internal distances. And the same is true for the type of apparent topological incorrectness reported in [9] among their top 400 distance constrained models, *“with the polypeptide chain passing through loops in a way that is, according to visual intuition, atypical of fully correct structures”*.

There are methods for protein structure comparison that do take topological features of proteins into account. Except for [15], none of these are alignment methods. Each of these methods first produces a global description of each protein chain in the form of a set of descriptor values. Protein chains are subsequently compared by comparing their descriptor values. The number of proteins and not the number of protein pairs dominate the calculation time. Hence, descriptor-based methods are very fast on large data sets as they neither need nor provide a structural alignment of sub structures. The first method to distinguish different threading of protein models was by calculating the writhe of each model [16]. The writhe is a signed measure of how coiled a space curve is and jumps discontinuously by $\pm\; 2$ when a curve passes through itself once. In [16] Michael Levitt reported a writhe separation between differently threaded models. However, two self-intersections may have canceling writhe jumps, and also continuous deformations change the writhe; thus, the writhe cannot separate folds by itself. The four distinct points involved in two self-intersections on an open directed curve form one of four orderings. Similarly, three self-intersections define 15 orderings. Generalizations of the writhe called Generalized Gauss integrals used in [17] are constructed such that at least one of these descriptors jumps discontinuously [18] in each of these 1 + 4 + 15 cases. The set of descriptors introduced in [19] also has this property. The generalized Gauss integrals are powerful enough to tell all fold classes in the protein classification CATH version 2.4 [17] apart, and they can be normalized to have desired metric properties [18]. However, as in the case of writhe, these types of descriptors are mathematically not powerful enough to tell any two configurations apart due to the dimension reduction taking place. As they apparently manage to separate protein folds, it is here more interesting that such global descriptors do not point out where along the backbone two structures are topologically different—the descriptor values are just significantly different. A similar remark holds for the topological descriptors based on fat graphs [20, 21], alpha shape [22] and on the average occurrences of all patterns of 3 crossings in planar projections of a protein backbone [19]. Not all descriptor-based methods are directly sensitive to topology. Examples are the method with the most accurate re-classification of protein structures reported, FragBag [23], and the distance excess-based descriptors [24]. The later method however presents an elegant solution to the problem of detecting (plastic) deformation as caused by mutations. The exact differential form underlying the descriptors makes them path independent such that they e.g. are invariant under any deformation of a loop in a protein that preserves the loops terminal points.

The effort put into detecting special topological features in a single protein structure illustrates the difficulty in detecting topological changes between pairs of protein structures. Knots in proteins [25, 26] have been studied for two decades [27, 28]. And lately also lassos [29] and links [30] in proteins have been investigated. The discrete nature of Monte Carlo sampling allows the protein backbone to pass through itself between samples, but in protein configuration space such a jump corresponds to a long and unlikely motion. Thus, in protein structure prediction, Monte Carlo sampling produces more knotted models than found in real proteins. The reader is encouraged to read the well told story [31] about the efforts taken both by modelers and assessors in the series of Critical Assessment of techniques for protein Structures Prediction (CASP) to avoid knotted and slip-knotted structures that in some cases have been the first model. The algorithm Pokefind [31] detects if a disk spanned by a shorter closed loop in a protein structure is penetrated by another part of the chain. In a protein model, this local non-protein like topological feature is likely to be a modeling error caused by the backbone passing through itself. Such topological model errors are apparently not detected by current structural alignment methods as [31] reports that neither the presence of knots nor of pokes in protein models show any significant correlation to the native-model distance measured by Global Distance Test (GDT). [31] concludes by suggesting adding Pokefind and Knotfind to the current metric. Their suggested method will tell if the number of knots and pokes differs between two protein structures. Hence, one would know that there is at least one topological obstruction between a knotted and an unknotted protein and at least one obstruction between a poked and a poke free structure. But topological obstructions separating differently folded structures will not be detected as long as knots and pokes are unchanged; most likely, by being absent in both structures. We conclude that no current algorithm can guarantee to find topological obstructions to a structural alignment and superposition of one protein structure onto another and present novel algorithms ProteinAlignmentObstruction for finding such obstructions and for evaluating how severe they are.

All protein backbones form open curves and are produced by extrusion. We here ignore the covalent links found when including disulfide bridges [30]. Hence, combining a complete unfolding of the first structure with a folding of the second from its complete unfolded stage gives a continuous self-avoiding path between two arbitrary protein structures. Thus, mathematically all protein chains share topology class and any sub-division of protein configuration space into folds depend on human judgement and is likely to depend on the specific structural biological context. Consider a pair of shoes with almost identical shoelaces except that the one contains a left-handed trefoil knot and the other a right-handed trefoil knot. In the space of immersions, they are very close, and any structural alignment program will find that, but in the space of embeddings, they are not close meaning no short self-avoiding morph between them exists. In structural biology terms, these two shoelaces share architecture, as the overall building blocks are very similar. They may or may not belong to different folds, also referred to as topology classes, depending if their different threading is recognized and considered important. But the folding paths have to be very different which may not be recognized. To address the difference between immersed and self-avoiding path-length ProteinAlignmentObstruction starts from the alignment and superposition provided by an alignment program and considers the linear interpolation between the two protein structures implied by the alignment score. First, it provides a novel measure of the amount of steric overlap caused by the morph. Next, it detects all self-intersections that occur during the interpolation and determines which are avoidable by moves similar to knot theory’s Reidemeister moves within a user defined tubular neighborhood of the original linear morph. Next, it solves the problem of avoiding the maximal number of self-intersections at the shortest additional morph-length. Either it finds a self-avoiding morph close to the original linear interpolation or it labels the remaining self-intersections essential, as they cannot be resolved sufficiently close to the original interpolation. Finally if wanted, the length of a self-avoiding morph that resolves these essential self-intersections but gets far from the original alignment is found. In this work, the focus is to make a fast algorithm that can detect steric and topological obstruction to a structural alignment and superposition of two protein structures at a calculation time compatible to that of a structural alignment program. The length of the self-avoiding morph provided here is at best a crude approximate of a shortest self-avoiding morph-length; a calculation that lies outside the scope of this paper as this author expect it to be too slow for practical application in pairwise protein structure comparison. Also it is not the aim to find geometrically or energetically plausible paths as e.g. considered in [32]. Conceptually, the main aim is to introduce homotopy of curve embeddings and Reidemeister moves to the field of computational structural biology as spoken for in [33].

The description of algorithms presented in the following section assumes no prior knowledge of knot theory and homotopy theory. It is somewhat lengthy, occasionally involved and may be skipped by readers not interested in the method development. Such readers should read the motivating example below and from there jump to the results section.

**Fig. 1:**
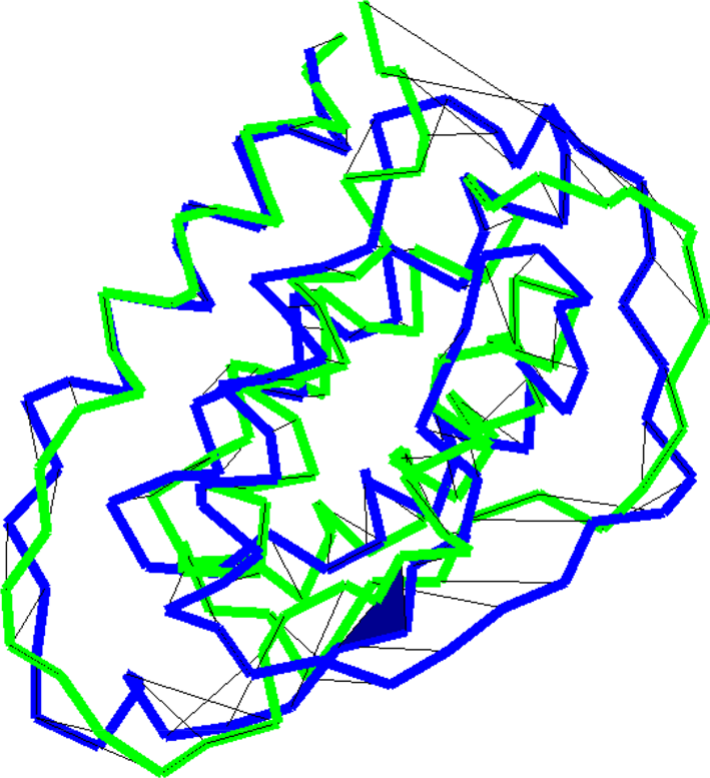
A self-intersection indicated by a black face in the morph between the alpha carbon curves of CATH2.4 domains 1csgA0 and 1jli00. The two domains share the homology-class 1.20.120.200

**Fig. 2:**
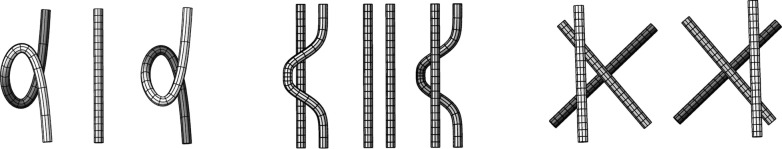
Reidemeister moves of types 0, 1, 2, and 3, denoted $\Omega _i,\, i=0,\dots ,3$. $\Omega _0$ deforms one arc without changing crossings (not shown). Left, $\Omega _1$ either adds one crossing to an isolated segment or deletes an isolated loop. In the middle, one of two parallel strands is slid either above or below the other by $\Omega _2$. To the right: $\Omega _3$ moves an arc across a crossing between two other arcs. By the usual right hand rule, the left most crossing in the picture of $\Omega _1$ is negative and the other crossing in this picture is positive meaning that if you choose a direction of traversing of the curve the streamlines fulfill the right hand rule

**Fig. 3:**
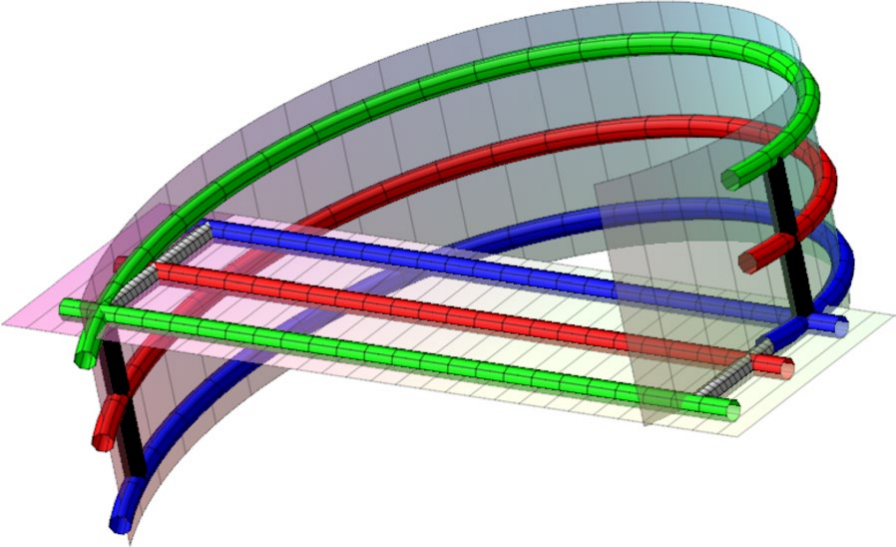
A space curve homotopy where the helical segment moves upwards and the straight segment moves inclined forward. The first intersection of the two segments is shown in blue and the second in green. Following the motion of the point of intersection on the blue helical segment between the two times of intersection, you get the black cylinder to the right-hand side. Inside this black cylinder the green helical segment can go down and around the intersection point of the two blue curve segments and back up to the green curve. Similarly, the gray cylinder to the right-hand side shows the motion of the first intersection point on the straight segment. Inside the gray cylinder, the green line segment may be altered to go back and around the intersection point. Hence, if the right hand side black and gray cylinders do not intersect other parts of the curve at a time between the two times of intersection, then we can alter all curves inside the two cylinders and postpone the intersection. Similarly, if the black and gray cylinders to the left-hand side do not intersect other parts of the curve between the times of the two intersections, then that intersection can be mover forward in time. Now move both intersections to happen at the mean of the two original intersection times (shown in red). The two red arcs combined with parts of the black and gray cylinders now form a closed loop. If a topological disc spanned by this loop avoids the remaining parts of the red curve, then two Reidemeister moves of type 2 can change the initial under-sliding of the helical segment to an over-sliding and avoid the two intersection points

## Methods

We start by estimating how similar two differently threaded tube structures fulfilling typical distance inequalities of alpha carbon atoms in proteins can be when measured by Root Mean Square Deviation (RMSD) [10], Global Distance Test - Total Score (GDT-TS) [7], and TM-score [11].

**Fig. 4:**
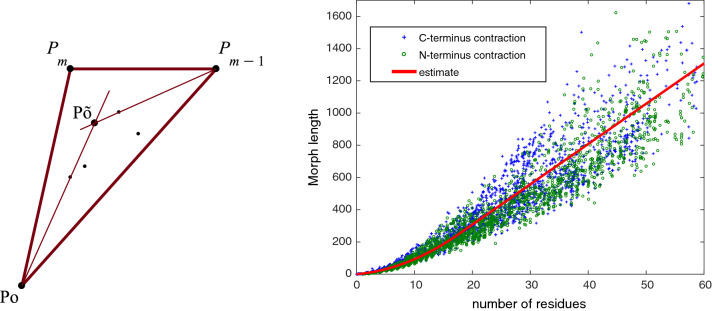
Left: the dots indicate intersections between triangle $Po,P_m,P_{m-1}$ with the remaining structure. The obstruction point $P{\tilde{o}}$ is chosen to minimize the path length *Po* to $P{\tilde{o}}$ to $P_{m-1}$. Right: the size of end-contraction morphs given as the sum of distance measured in Ångström traveled by all residues are shown as a function of the number of residues contracted. N- and C-terminus contraction seem similar, and their common estimate is explained in the text

**Fig. 5:**
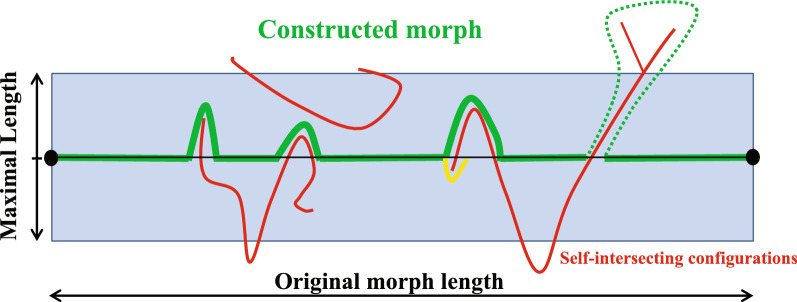
This figure illustrates the configuration space of a chain. The circles at the extreme left and right indicate the two aligned structures. The original linear interpolation is the black thin line segment between them. This line segment in the configuration space has six self-intersections where it crosses the thin red curves representing self-intersecting configurations. Each Reidemeister move may involve ${\text {MaxLength}}$ residues and can at most deviate a fixed distance, roughly $1/4{\text {MaxLength}}^2$, from the original linear interpolation. From left to right, the first self-intersection is avoidable by an $\Omega _1$-move and the next two by an $\Omega _2$-move. If the fourth self-intersection is removed by the short thick light colored $\Omega _1$-move, then the fifth cannot be removed. Hence, the $\Omega _2$-move is preferred. The last self-intersection involves too many residues to remove, is denoted essential, and the length of the dotted end-contraction avoiding it is calculated

**Fig. 6:**
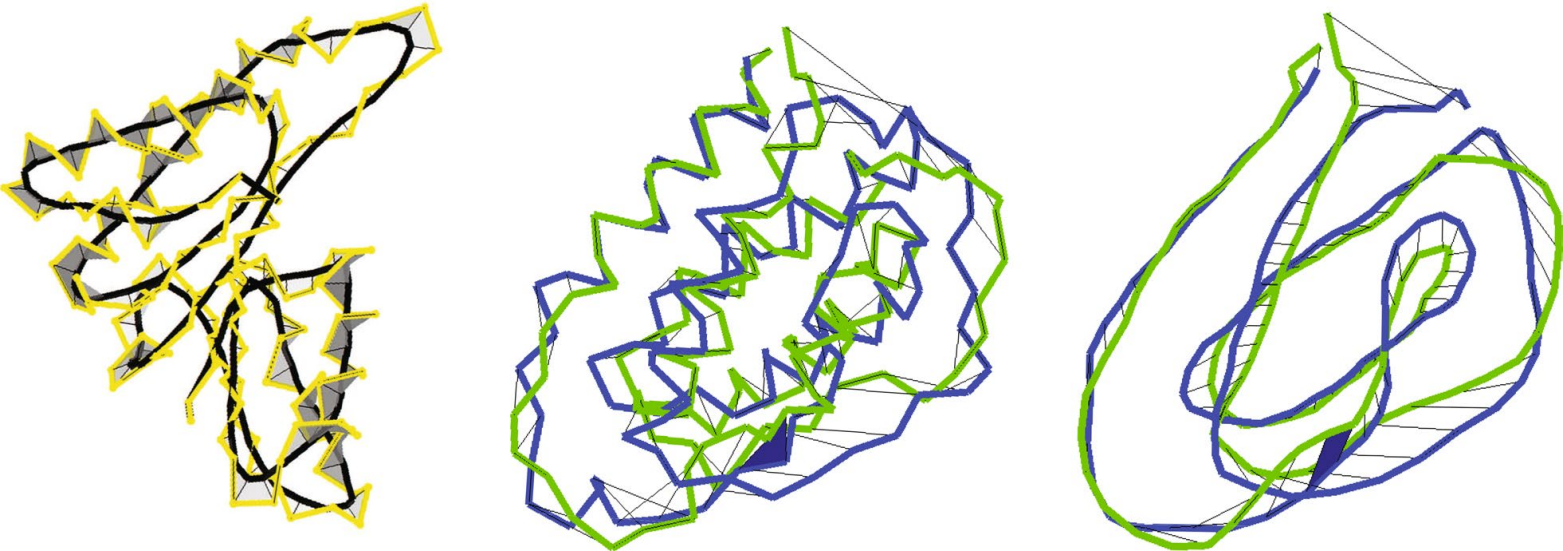
Left: the alpha carbon trace of CATH domain 1emd01 and its self-intersection free interpolation with its smoothened black curve. Center and right: the interpolation between CATH domains 1csgA0 and 1jli00 alpha carbon and smoothened curves respectively. The two domains share the homology-class 1.20.120.200. Both interpolations have one self-intersection indicated by a black face. In both cases it may be circumvented by a large $\Omega _1$ move. If the user does not allow such large rearrangements, this self-intersection is called essential and the size of an end-contraction avoiding it is calculated. As the alpha carbon RMSD is 5.6Å this is an example that a change in topology that seems to demand quite different folding pathways may happen for a smaller RMSD and for homologous chains

**Fig. 7:**
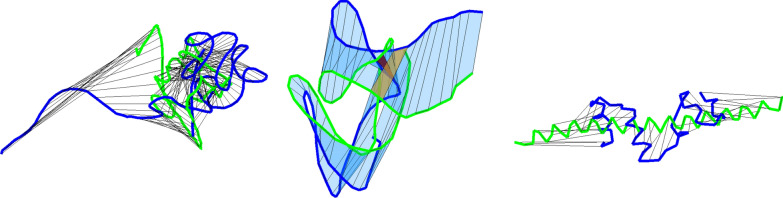
Left: the self-intersection free linear interpolation between the smooth backbones of the un- and phosphorylated forms of Odhl (NMR structures 2KB3 and 2KB4). Center: the interpolation between two configurations of the SH3 domain given by the A chains of 1AOJ and 1I0$\hbox {C}^*$ has a self-intersection between line segments approximately 10 and 25 residues from the C-terminal. Right: the interpolation between the 52 residue long helix 1IK7 A $\hbox {chain}^*$ and its aligned part of the 102 residue folded 1D2Z C chain is self-intersection free

**Fig. 8:**
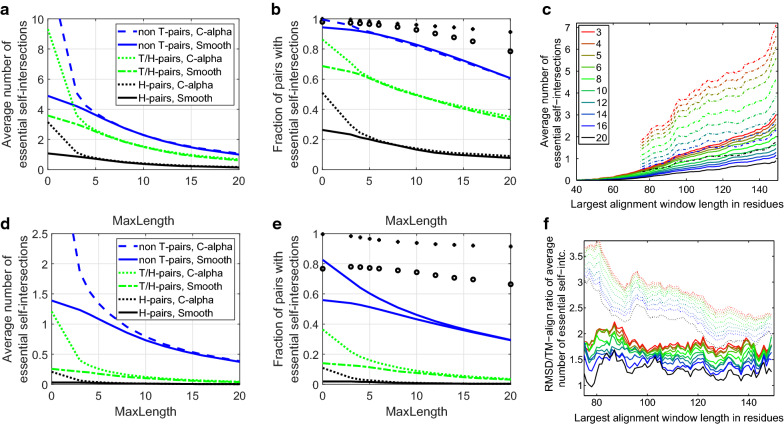
**a**: the average number of essential self-intersections found in the global RMSD alignment for pairs of CATH2.4 domains sharing homology class (H-pairs), sharing topology class but not homology class (T/H-pairs), or from distinct topology classes (non T-pairs). **b**: the fraction of pairs with essential morph self-intersections based on the global RMSD alignments. The plus signs show the fraction of the pairs with an essential self-intersection for the smooth curves that also have one for the alpha carbon curves. The circles show for the smooth curves the fraction of pairs with an essential self-intersection for the TM-alignment that also have one for the global RMSD alignment. **c**: the average number of essential self-intersections for smooth curves shown as function of the length of the largest aligned window and for ${\text {MaxLength}}$-values given in the legend. The graphs are smoothened by averaging over alignment windows of length between i – 2 and i + 2. Global RMSD alignments are dashed and TM-alignments in solid lines. **d** , **e**: as (**a**, **b**) but for the TM-alignment. In (**e**) the circles show the fraction of pairs with an essential self-intersection for the global RMSD alignment that also have one for the TM-alignment restricted to the 5% cases where $AWF\ge 0.9725$. **f**: the average number of essential self-intersections for the RMSD alignment divided by the same number for the TM-alignment and shown as function of the largest aligned window length. ${\text {MaxLength}}$ is color-coded as in (**c**). Dashed curves are for all alignments and solid curves for the 5% cases where $AWF\ge 0.9725$

**Fig. 9:**
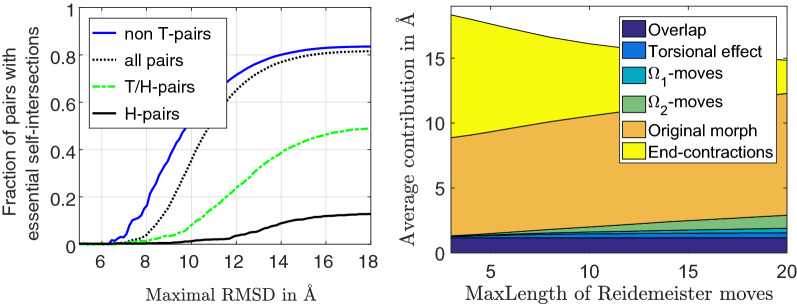
Left: the frequency of morphs with essential self-intersections for pairs with RMSD below a given maximal RMSD. The ${\text {MaxLength}}$ of $\Omega _1$ and $\Omega _2$ moves is set to 10. Right: the average contributions to the morph length for all intra T morphs shown as function of ${\text {MaxLength}}$. The absolute value of the signed sum of sign changes represent the torsional effect; the remaining contributions are average values per residue

### A motivating example

Alpha carbon atom neighbors along the backbone are usually $3.8\AA$ (Ångström) apart. Otherwise, alpha carbon pairs are rarely less than $4.5\AA$ apart. Imagine a symmetrical crossing in the *xy*-plane where the over-crossing part of a chain has heights (measured on the *z*-axis) 0, 0, 1.25, 2.5, 2.5, 1.25, 0, 0Å. Similarly, the under-crossing part follows the *y*-axis at heights 0, 0, – 1.25, – 2.5, – 2.5, – 1.25, 0, 0Å. Consider two larger structures that are identical except that the above over- and under-crossings are interchanged. The Euclidean motion of the optimal superposition of two structures is close to the identity, which we assume for simplicity. The total motion is thus given by 4 residues traveling $5\AA$ and 4 residues traveling $2.5\AA$. For an *n*-residue structure we find a topology change (tc) for

$$\begin{aligned}   RMSD_{n}^{{tc}} \; \le& \sqrt {\frac{{4(5{\AA})^{2}  + 4(2.5{\AA})^{2} }}{n}}  < \frac{{12{\AA}}}{{\sqrt n }} \hfill \\ &  GDT - TS_{n}^{{tc}} \; \ge \frac{1}{4}\sum\limits_{{m = 1,2,4,8}} {\text{aligned\;residues\;within\;m{\AA}}}  \hfill \\   &\frac{1}{4}\left( {\frac{{n - 8}}{n} + \frac{{n - 8}}{n} + \frac{{n - 4}}{n} + \frac{n}{n}} \right)\; =& \;1 - \frac{5}{n} \hfill \\   &TM_{n}^{{tc}}  \ge \frac{1}{n}\sum\limits_{{i = 1}}^{n} {\frac{1}{{1 + \left( {\frac{{d_{i} }}{{d_{0} }}} \right)^{2} }}}  \hfill \\    =& \frac{{n - 8}}{n} + \frac{4}{n}\frac{1}{{1 + \left( {\frac{5}{{d_{0} }}} \right)^{2} }} \hfill \\   & > 1 - \frac{4}{n},for\;n \ge 100, \hfill \\  \end{aligned} $$

where $d_0=1.24(n-15)^{1/3}-1.8$. For $n=100$ we find $RMSD^\text {tc}_{100}=1.2\AA$, $GDT-TS^{\text {tc}}_{100}=0.95$ and $TM^\text {tc}_{100}=0.96$. These scores are typical for very similar structures despite the constructed topology change. The persistence length of native proteins may require longer deformations than used in this example; but in computational modeling when, e.g., trying to minimize the distance between a model and a native structure one may get close to this example. Figure 1 shows a similar crossing change between two native 112 residue structures aligned with global RMSD $5.6\AA$ and TM= 0.60. Before describing a method to detect such topological changes during a linear interpolation between structures we start with an algorithm for detecting steric clashes under a linear interpolation.

### Measuring protein-protein overlap under a linear interpolation

Let two protein chains be represented by the coordinates of their alpha carbon atoms $P_0=\bigl [p_0^1 \dots p_0^n\bigl ]$ and $P_1=\bigl [p_1^1 \dots p_1^n\bigl ]$ each with index starting at the N-terminus. Assume they are placed in optimal superposition by RMSD or any other alignment method. We study the linear interpolation where each point $p^i(t)=(1-t)p_0^i+tp_1^i$ traverses the straight line segment connecting the *i*’th alpha carbon in each of the two structures and the parameter $t\in \bigl [0,1\bigl ]$ may be thought of as a time parameter. We want to check if typical protein distance constraints are violated during the interpolation and quantify how much the protein overlaps itself. For this, we first find the minimal distance between $p^i$ and $p^j$ during the interpolation. Let $p^{i,j}_0=p_0^j-p_0^i$, $p^{i,j}_1=p_1^j-p_1^i$ and denote $\bigl \Vert p^{i,j}_0\bigl \Vert =a$, $\bigl \Vert p^{i,j}_1\bigl \Vert =b$, and $p^{i,j}_0\cdot p^{i,j}_1=ab\cos {\theta }$. For all *t* we have

$$\begin{aligned} d^2_{i,j}(t)= & {} \bigl \Vert p^j(t)-p^i(t)\bigl \Vert _2^2=\bigl \Vert (1-t)p^{i,j}_0+tp^{i,j}_1\bigl \Vert {_2^2}\\=\; & {} (1-t)^2a^2+t^2b^2+2t( 1-t)ab\cos {\theta }. \end{aligned}$$

The global minimum of $d^2_{i,j}$ is found for $t^*=\frac{a^2-ab\cos {\theta }}{a^2+b^2-2ab\cos {\theta }}$ and equals

$$\begin{aligned} m= & {} \frac{\sin {\theta }a^2b^2}{a^2+b^2-2ab\cos {\theta }}=\frac{\bigl \Vert p_1^{i,j}\times p_0^{i,j}\bigl \Vert _2^2}{\bigl \Vert p_1^{i,j}- p_0^{i,j}\bigl \Vert _2^2}\\= & {} \frac{\bigl \Vert \bigl (p_1^{i,j}- p_0^{i,j}\bigl )\times p_0^{i,j}\bigl \Vert _2^2}{\bigl \Vert p_1^{i,j}- p_0^{i,j}\bigl \Vert _2^2}. \end{aligned}$$

The last equation shows that the fraction is bounded by $\bigl \Vert p_0^{i,j}\bigl \Vert _2^2$ and by symmetry also by $\bigl \Vert p_1^{i,j}\bigl \Vert _2^2$. Setting ${\tilde{t}}=\bigl \{0$, if $t^*<0$; $t^*$, if $0\le t^*\le 1$; and 1, if $1<t^*\bigl \}$, the minimum of $d^2_{i,j}$ for $t\in [0,1]$ denoted $d^\text {2,interpolation}_{i,j}=d^2_{i,j}({\tilde{t}})$. If $\bigl \Vert p_1^{i,j}- p_0^{i,j}\bigl \Vert _2^2$ is vanishing $t^*$ is ill-defined; but then $d^2_{i,j}$ is constant and this value is returned. From a set of native protein structures, we estimate the shortest possible distances between alpha carbons *i* and *j* to be $d_{min}(|i-j|)=$ 2.8, 4.5, 3.86, 3.47, 3.52, 3.48, 3.6Å for $|i-j|=1,\dots ,7$ and $d_{min}(|i-j|)=3.7\AA$ for $|i-j|>7$. The overlap, $\text {overlap}_{i,j}$, is thus the max of $d_{min}(|i-j|)-d^\text {interpolation}_{i,j}$ and 0(zero). The mean overlap is given by ${\text {MeanOverlap}}\big ( P1,P2\big )=\frac{1}{n}\sum _{i<j} \text {overlap}_{i,j}$. Especially in $\alpha$-helices the distance between neighbouring alpha carbon atoms may be shortened substantially during the interpolation. Therefore $d_{min}(1)=2.8$ is chosen small to avoid large contributions from local deformations. If both protein structure $P_0$ and $P_1$ are overlap free, $\text {overlap}_{i,j}$ is given by the max of $d_{min}(|i-j|)-\sqrt{m}$ and 0(zero) if $0<t^*<1$ and 0(zero) otherwise. To be able to handle models not fulfilling basic distance constraints, we do not make this assumption.

It is a priory not clear how to penalize protein-protein overlap. Here, we have chosen to penalize pairwise overlap linearly.

### Topology check of a protein under a linear interpolation

We now leave the volumetric point of view and turn to a purely topological point of view. Given two superimposed protein structures, we consider the linear interpolation of the piecewise linear curve connecting the alpha carbons. Let *P* denote the piecewise linear curve through all points $p_i$, $i=1,\dots ,n$. That is $P_a=p_i$ for $a=1,\dots ,n$, but $P_a$ is defined for all $a\in [1,n]$. The linear interpolation between two piecewise linear curves $P^0$ and $P^1$ is given by $P_a(t)=(1-t)P^0_a +tP^1_a$ for $t\in [0,1]$ and $a\in [1,n ]$.

We want to detect all self-intersections of the backbone curve during the linear interpolation. Furthermore, we want to identify all self-intersections we can avoid by changing the initial morph locally by rearranging at most a user defined number of residues. The remaining self-intersections are expected to do essential changes to the fold, e.g., as avoiding them require larger rearrangements than allowed by the user. To do this, we borrow some notation from knot theory concerning knots on closed curves in 3-space. For a knot in 3-space a planar projection with over and under crossings indicated is called a knot diagram if all crossings are isolated. Hence, at most two points on the knot are allowed to be projected to the same point. When changing the plane of projection the crossings will move, crossings may disappear, new crossings may appear, and in the planar projection existing crossings may pass through each other. The same happens to the planar projection when deforming the knot through a self-avoiding morph. A fundamental result found in any text book on knot theory states that two closed curves share knot type, i.e., the one may be morphed into the other through imbeddings by a ambient isotopy if and only if their knot diagrams are connected by a finite series of Reidemeister moves, shown in Fig. 2, plus deformations not changing crossings. Crossings of a knot may be associated with a sign using the usual right-hand rule explained in the same figure. Note, that changing the direction of traversing a knot does not change the sign of the crossing. The sign of a crossing comes from the orientation of 3-space and is for line segments $P_i P_{i+1}$ and $P_j P_{j+1}$ given by the sign of the determinant $\det _{ij}=\det \bigl ( P_{i+1}-P_{i}\, P_{j+1}-P_{j}\, P_{i}-P_{j} \bigl )$.

#### Detecting transversal self-intersections

We consider only so-called transversal self-intersections where two line segments actually go through each other and consequently the sign of their crossing changes at the time of the self-intersection. Algorithmically, we thus first find isolated real roots $t^*\in [0,1]$ for the 3’rd order polynomial

$$\begin{aligned}&{\text {det}}_{ij}\bigl (t\bigl ) = \\& \det \bigl ( P_{i+1}(t)-P_{i}(t)\, \quad P_{j+1}(t)-P_{j}(t)\, P_{i}(t)-P_{j}(t)\bigl ). \end{aligned}$$

For each of these roots, $t^*\in [0,1]$, the line segments $P_i(t^*)P_{i+1}(t^*)$ and $P_j(t^*)P_{j+1}(t^*)$ lie in a plane and intersect only if the planar problem

$$\begin{aligned}&(1-s_i)P_{i}(t^*)+s_iP_{i+1}(t^*)\\&\quad =(1-s_j)P_{j}(t^*)+s_jP_{j+1}(t^*) \end{aligned}$$

has a solution with $s_i,s_j\in [0,1]$. If a self-intersection is found, we store the interpolation parameter $t^*$, the curve parameters of the self-intersection $a=i+s_i$ and $b=j+s_j$, together with the crossings sign change given by the sign of the derivative of $\det _{ij}(t)$ at $t^*$. By checking all pairs of line segments we get a list of self-intersections with data $( a_k,b_k,sign_k,t^*_k)$, where *k* is an arbitrary index.

A self-intersection between line segments $P_i P_{i+1}$ and $P_j P_{j+1}$ requires that at least some of the spheres centered at $P_i$, $P_{i+1}$, $P_j$ and $P_{j+1}$ are overlapping during the interpolation as calculated in the previous section. The self-intersection check may be skipped if the alpha carbon neighbors are less than $4\AA$ apart and ${\text{overlap}}_{{i,j}}  + {\text{overlap}}_{{i,j + 1}}  + {\text{overlap}}_{{i + 1,j}}  + {\text{overlap}}_{{i + 1,j + 1}} \; < \;2.6{\AA}$. The pre-computed overlap gives an efficient filter before the self-intersection check, but one cannot tell a nearby passing from a self-intersection by the overlap values.

#### Locally avoidable self-intersections

Some morph self-intersections make a significant change to a proteins fold, while others do not. In the mathematical notion of topology however all protein chains are topologically equivalent as they all may be refolded e.g. into one long beta strand. Algorithmically such a morph may be constructed by a blow-up technique. A distinction between significant and insignificant fold changes can thus only be decided by human judgement and may differ depending on the application at hand. Here, we search for insignificant self-intersections that we can resolve by local changes of the morph and quantify how much longer the resulting morph is.

Imagine the left- and right-handed loops in Fig. 2 aligned on top of each other. The linear interpolation between the two structures will have a self-intersection. Loops are the most flexible parts of proteins and such a self-intersection is a minor structural change as we now explain. Mathematically this self-intersection can be removed from the morph as this structural change may be performed by two Reidemeister moves of type one, denoted $\Omega _1$. We have used that the projection of the loop in Fig. 2 is isolated from the rest of the projected curve. In a projection of a globular protein structure, it is likely that the projection of other parts of the backbone pass through the projected loop. Hereby a longer series of Reidemeister moves may be needed to connect the projections of the two loops. To avoid this algorithmic complexity we choose to make a 3-dimensional analogue of Reidemeister moves. We want to know if we can remove a self-intersection by just changing the morph of the arc forming the loop while leaving the rest of the curve and its morph unchanged. We therefore say that a self-intersection $( a_k,b_k,sign_k,t^*_k)$ is locally $\Omega _1$-removable if the topological disk given by all the triangles connecting two consecutive points of the loop

$$    P_{{a_{k} }} \;\left( {t_{k}^{*} } \right),P_{{ciel(a_{k} )}} \left( {t_{k}^{*} } \right), \ldots ,P_{{floor(b_{k} )}} \left( {t_{k}^{*} } \right),P_{{b_{k} }} \left( {t_{k}^{*} } \right)\; = \;P_{{a_{k} }} \left( {t_{k}^{*} } \right) $$

to the center of mass of all these points is disjoint from all the other line segments of the $t^*_k$-curve $P_{1}(t^*_k),\dots , P_{floor(a_k)}(t^*_k)$ and $P_{ciel(b_k)}(t^*_k),\dots , P_{n}(t^*_k)$. In this case, one can freely deform the loop in a neighborhood of the topological disk without intersecting the rest of the curve. One possible morph that avoids the self-intersection contracts the loop almost to its center of mass where there is room to throw one arc around the loop. However, for less complicated loops one would simply deform the loop by rotating it around the line through the point of self-intersection and the center of mass of the loop. We therefore penalize a locally $\Omega _1$-removable self-intersection by a price ${\mathscr {P}}1$ equal to twice the sum of the distances from the points of the loop to this line. The self-intersection shown in Fig. 1 may be removed by an $\Omega _1$ move involving a large part of the protein. We therefore let the user decide the maximal accepted backbone length, denoted ${\text {MaxLength}}$ involved in the alternate morph. Only self-intersections with $b_k-a_k\le {\text {MaxLength}}$ are tested for being $\Omega _1$ removable. In a planer projection into a knot diagram the curve segment from $a_k$ to $b_k$ need neither be simple nor isolated from the remainder of the projected curve. The constructed $\Omega _1$-move is operating on a 3-dimensional curve. It is therefore not a real Reidemeister move that acts on 2-dimensional knot diagrams. The $\Omega _1$-move need not project to a Reidemeister 1 move for two main reasons. Firstly, the 3-dimensional loop need not project to a simple curve. For larger ${\text {MaxLength}}$ the loop may even be knotted. Secondly, in the knot diagram the projection of the loop is not likely to be isolated from the remaining projection. Thus the 3-dimensional $\Omega _1$-move may involve many Reidemeister moves in a knot diagram and can be seen as a meta move in the context of knot diagrams.

If a closed double stranded DNA undergoes the self-intersection considered here, it will change its writhe and hereby also the linking number between the two strands by plus or minus two [34]. To feel this coupling between writhe and twisting, hold a length of torsional stiff wire in the shape of a left-handed loop such that it is in self-contact almost forming a self-intersection. By rotating both ends of the wire, you can force the wire to morph into a right-handed loop. That is, a morph avoiding the self-intersection requires two full rotations around the backbone. Proteins are in principle free to do full rotations around the backbone. One may choose not to penalize the discontinuous writhing caused by $\Omega _1$ loops but we offer the possibility.

Most self-intersections are not $\Omega _1$-removable. A significant speed up of the calculation time is obtained by: First, move the origin of the coordinate system to the center of mass of the loop, which then is a vertex in all the triangles of the topological disc. Then to sort all line segments by increasing distance from the origin to the center of the line-segments. Finally, to check all line-segment triangle pairs for intersection and stop either when an intersection is found or when the triangle inequality between the disk and line segments implies that an intersection is impossible.

The local zigzag shape of general alpha carbon curves often make self-intersections occur in pairs during a morph. Such a pair corresponds to interchanging over and under sliding in Reidemeister moves of type two, $\Omega _2$, shown in the middle of Fig. 2. The two crossings, $( a_j,b_j,sign_j,t^*_j)$ and $( a_k,b_k,sign_k,t^*_k)$, in an $\Omega _2$ move have opposite signs and both change sign during transversal self-intersections. Without loss of generality, we may assume that $t^*_j\le t^*_k$. Consider first the case where $t^*_j= t^*_k$. At time $t^*_k$ the two arcs connecting the two points of self-intersection form a closed curve. If a topological disc spanned by this closed curve is disjoint from the remaining parts of the curve then the two self-intersections can be removed essentially by performing two Reidemeister moves of type two close to this disc. You may construct this such that one of the moves occurs before the time $t^*_k$ and one after. In the general case where $t^*_j\le t^*_k$ it is sufficient that a topological disk whose boundary includes the two arcs is isolated for some $t\in \big [t^*_j; t^*_k\big ]$, see Fig. 3. To close the curve and to have room for a morph avoiding the two self-intersections we have to add the line segments traversed by the self-intersection points in the time interval $t^*_j\le t\le t^*_k$. In practice we check if the topological disc connecting the loop for $t^*_{average}=(t^*_j+t^*_k)/2$ to its center of mass is disjoint from the remainder of the curves for $t=t^*_{average}$ and if the additional line segments traversed by the self-intersection points are disjoint from the remainder of the curve in the time interval $t^*_j\le t\le t^*_k$. See Fig. 3. The first check is speeded up as in the case of the $\Omega _1$ move. If the backbone length of the loop is shorter than the user-defined ${\text {MaxLength}}$, i.e., if $b_j-a_j+b_k-a_k\le {\text {MaxLength}}$, and no obstructions are found we say that $( a_j,b_j,sign_j,t^*_j)$ and $( a_k,b_k,sign_k,t^*_k)$ form a locally $\Omega _2$-removeable loop and penalize it by price ${\mathscr {P}}2$ twice the sum of the distances from the points of the loop to the line connecting the two points $P_{a_j}(t^*_{average})$ and $P_{a_k}(t^*_{average})$.

As in the case of $\Omega _1$ moves, an $\Omega _2$ move acts on space curves. When projected to a knot diagram, an $\Omega _2$ move may require several ordinary Reidemeister moves and may thus be thought of as a meta move of knot diagrams. The sum of the crossings signs is unchanged by an $\Omega _2$ move and there is thus no net discontinuous contribution to the writhe of the curve. E.g., the linking number of double stranded DNA undergoing an $\Omega _2$ move is unchanged and there is therefore no torsional penalty involved.

#### Essential self-intersections

Let $\{v_i\}$ denote all the self-intersections. Some are $\Omega _1$-removable at price ${\mathscr {P}}1(v_i)>0$. Some pairs $[v_i,v_j]$ are $\Omega _2$-removable at price ${\mathscr {P}}2(v_i,v_j)>0$. We want to remove as many self-intersections as possible at the lowest possible cost when being restricted to removing each $v_i$ at most once. These two problems may be solved simultaneously by one maximal weight matching problem as we now explain. We will denote the self-intersections left after this process a set of essential self-intersections of the morph. Consider the $v_i$’s as vertices in a graph and let $\Omega _1$ denote the $\Omega _1$-removable subset. Let the constant $\varepsilon$ equal one over twice the sum of the prices of all $\Omega _1$ and $\Omega _2$ moves. Each vertex is associated a weight

$$\begin{aligned} w_v(v_i)=\left\{ \begin{array}{ll} \varepsilon {\mathscr {P}}1(v_i), &{} \text {if} \; v_i\in \Omega _1 \\ 1, &{} \hbox {else.} \end{array} \right. \end{aligned}$$

We want to remove as many vertices as possible. Hence, each vertex in $\Omega _1$ will be removed by an $\Omega _1$ move unless it’s more favorable to let it take part in an $\Omega _2$ move. Hence, we only need to consider how to use the $\Omega _2$ moves. For this, let each $\Omega _2$ move be represented by an edge, *e*, connecting vertices $v_i$ and $v_j$ and associate this edge with the weight

$$\begin{aligned} w(e)=w_v(v_i)+w_v(v_j) - \varepsilon {\mathscr {P}}2(v_i,v_j). \end{aligned}$$

Finally, let *K* be the number of vertices not in $\Omega _1$, denote the total cost of the $\Omega _1$ moves by ${\mathscr {P}}1_{all}=\sum _{v_i\in \Omega _1} {\mathscr {P}}1(v_i)$, and recall that a matching is a subset $E'$ of all the edges that uses each vertex at most once. Let $N(E')$ denote the number of vertices left after performing the $\Omega _2$ moves specified by $E'$ followed by all the possible $\Omega _1$ moves and let ${\mathscr {P}}(E')$ be the sum of the prices of all the $\Omega _1$ and $\Omega _2$ moves used. We claim that for each matching $E'$

$$\begin{aligned} N(E')+\varepsilon {\mathscr {P}}\bigl (E'\bigl )=K+\varepsilon {\mathscr {P}}1_{all} -\sum _{e\in E'} w(e) \end{aligned}$$

and denote this quantity by $A\bigl (E'\bigl )$. By this claim a maximal weighted matching $E^*$ maximizes $\sum _{e\in E'} w(e)$ and thus minimizes the left-hand side. By construction $0\le \varepsilon {\mathscr {P}}(E')\le \frac{1}{2}$ and hereby $N(E^*)$ is minimized and ${\mathscr {P}}(E^*)$ is minimized subject to the constraint that $N(E^*)$ is minimal, solving both problems at once.

We find it most instructive to prove this claim by induction on the cardinality of $E'$ and start with the case $E'=\emptyset$ where all $\Omega _1$ moves are performed and the claim is trivial. Consider a matching $E''$ where $e_k\in E''$ and the induction claim is true for $E'=E''-\{e_k\}$. Then

$$\begin{aligned} A\bigl (E''\bigl )&=K+\varepsilon {\mathscr {P}}1_{all} -\sum _{e\in E''} w(e)\\&= K+\varepsilon {\mathscr {P}}1_{all} -\sum _{e\in E'} w(e)-w(e_k)\\&= N(E')+\varepsilon {\mathscr {P}}(E')-w(e_k), \end{aligned}$$

where the last equality follows by induction. We use the shorthand ${\mathscr {P}}2(e_k)$ for the price ${\mathscr {P}}2(v_i,v_j)$ of the edge $e_k$ between the two vertices $v_i$ and $v_j$.

If $v_i,v_j \notin \Omega _1$ then $w(e_k)=2-\varepsilon {\mathscr {P}}2(e_k)$ and $A(E'')=N(E')-2 +\varepsilon \bigl ( {\mathscr {P}}(E') +{\mathscr {P}}2(e_k)\bigl )$. As two additional vertices are removed at the cost ${\mathscr {P}}2(e_k)$ we find $A(E'')=N(E'')+\varepsilon {\mathscr {P}}(E'')$.

If $v_i\in \Omega _1$ and $v_j \notin \Omega _1$ then $w(e_k)=\varepsilon {\mathscr {P}}1(v_i) +1-\varepsilon {\mathscr {P}}2(e_k)$ and $A(E'')=N(E')-1 +\varepsilon \bigl ( {\mathscr {P}}(E') -{\mathscr {P}}1(v_i)+{\mathscr {P}}2(e_k)\bigl )$. Here one additional vertex, $v_j$, is removed and the change of cost is ${\mathscr {P}}2(e_k)-{\mathscr {P}}1(i)$ as $v_i$ not is removed by a $\Omega _1$ move but by the $\Omega _2$ move $e_k$. Thus $A(E'')=N(E'')+\varepsilon {\mathscr {P}}(E'')$.

Finally, if $v_i,v_j\in \Omega _1$ then $w(e_k)=\varepsilon {\mathscr {P}}1(v_i) +\varepsilon {\mathscr {P}}1(v_i)-\varepsilon {\mathscr {P}}2(e_k)$ and $A(E'')=N(E')+\varepsilon \bigl ( {\mathscr {P}}(E') -{\mathscr {P}}1(v_i)-{\mathscr {P}}1(v_j)+{\mathscr {P}}2(e_k)\bigl )=N(E'')+\varepsilon {\mathscr {P}}(E'')$ as no additional vertices are removed and the cost is changed by using the $\Omega _2$ move $e_k$ instead of the two $\Omega _1$ moves. This completes the proof of the claim.

We use the Matlab implementation [35] based on [36]. In practice, this calculation uses only a small fraction of the computation time and may be considered efficient. We also implemented a greedy algorithm that searches for locally contractible $\Omega _2$ moves sorted by increasing backbone length between the two points of self-intersection and removes the ones found. Usually considerable fewer candidate $\Omega _2$ moves have to be checked. Hence, the greedy algorithm is faster but in some cases it cannot remove as many self-intersections as the optimal method based on maximum weighted matching in graphs. There may be several sets with a minimal number of essential self-intersections, but in most cases the real valued prices of $\Omega _1$ and $\Omega _2$ moves will result in a unique cheapest minimal set. However, for some morphs a slight change in these prices will change the selected minimal set. For more complicated morphs we therefore stress not to emphasise which self-intersections are denoted essential but solely to report their cardinality.

### End-contractions

When a self-intersection is not locally removable, we construct a morph using a standard strategy for unknotting ropes or shoelaces namely: while keeping most of the rope fixed, pull the rope until a terminus gets free of the loop restraining it. That is, a self-intersection $( {{a_j},\;{b_j},\;{sign_j},\;{t_j^*}})$ may be avoided by N- or C-terminus contraction. An N-terminus contraction first moves all points upstream of $P_{a_j}(0)$ to this point for interpolation parameter $t=0$, then uses the corresponding restriction of the original morph from $t=0$ to $t=1$, and finally does a reversed end-contraction on the protein structure for $t=1$. If the end-contraction is restricted to be performed along the backbone path, $P_1\dot{(}0)$ is moved a total distance of approximately $a_j 3.8\AA$, $P_2$ is moved approximately $(a_j-1)3.8\AA$ etc. The total morph length is therefore quadratic in the distance to the terminus. In general, the following algorithm illustrated on an N-terminus contraction finds a much shorter end-contraction.

The end-contractions is performed on the original protein structures, and we omit writing the constant interpolation parameter *t* = 0 or 1. That is $P{a_j}$ is shorthand for either $P_{a_j}(0)$ or $P_{a_j}(1)$. Let the first obstruction point be $Po=Pa_j$ and let $m=floor(a_j)$. We have to move the entire curve $P_1,\dots ,P_m$ to the point $P_{a_j}$. $P_m$ may always be moved along the straight line to $P_{a_j}$. If the triangle with corners $Po, P_m, P_{m-1}$ is disjoint from the curve downstream of $P(a_j)$ the shortcut from $P_{m-1}$ to *Po* may be used. Otherwise a new obstruction point $P{{\tilde{o}}}$ inside the triangle is chosen such that the length of the path *Po* to $P{{\tilde{o}}}$ to $P_{m-1}$ is minimized and all downstream line segments are avoided. See Fig. 4. The algorithm is greedy and accepts the path from $P{{\tilde{o}}}$ to $P_{a_j}$ as the shortest found and all upstream points will use this as part of their path to $P_{a_j}$. The last encountered obstruction point is moved to $P{\tilde{o}}$. Then this shortening of the end-contraction is continued for all the upstream line segments. The cost of avoiding the self-intersection $( a_j,b_j,sign_j,t^*_j)$ is thus the total distance traveled by the points in the end-contraction both for $t=0$ and for $t=1$ together with *m* times the distance traveled by $P(a_j)$ in the original linear interpolation, because all the *m* contracted points travel this distance.

If $a_1\le a_2\le ,\dots ,\le a_n$ and $b_1,b_2,\dots ,b_n$ are the parameter values of the essential self-intersections the combined smallest N and C terminus end-contractions removing them all solve:

$$\begin{aligned}  \min _{{1\; \le \;a\; < \;b\; \le \;L}} {\text{ N - Contraction}}(a) + {\text{C - Contraction}}(b)  \\ \quad{\text{s.t.}}\,a_{i} \; \le a\;or\;b \le b_{i} \;{\text{for}}\;{\text{all}}\;i=1,\dots ,n. \end{aligned}$$

If no ${\text {N-contraction}}$ is used $a=1$ and $b=\min (b_1,b_2,\dots ,b_n,L)$ in order to remove all the self-intersections. If $a=a_1$ the first self-intersection is removed from the N-terminus end and $b=\min (b_2,\dots ,b_n,L)$, removes the remaining self-intersections. Thus in general $a=a_j$ and $b=\min (b_{j+1},\dots ,b_n,L)$ fulfill the constraints and we only have to check $n+1$ cases to find this minimum. To speed this calculation up, we use an estimate of the end-contraction based on the observation that the distance traveled by residues up to 17 residues away from the self-intersection point grow roughly linearly to 25Å and is fluctuating around 25Å when further apart along the backbone. This gives a reasonable estimate of the sizes of the chosen end-contractions as shown on Fig. 4. The end-contractions are the computationally most costly part of all the calculations. As they only serve to quantify the distance between essentially different folds, we offer the possibility to omit this calculation and instead use the estimated size of the end-contractions. Finally, we offer the possibility to consider a self-intersection as $\Omega _1$-removable if it is sufficiently close to a protein terminus. One natural choice is to set the longest allowed distance from the point of self-intersection to an end to half of the user-defined ${\text {MaxLength}}$. In case a self-intersection also is $\Omega _1$-removable the price is set to the minimum of the $\Omega _1$ price and the end-contraction price. Figure 5 illustrates the full self-avoiding path that lies in a tubular neighborhood of the original linear interpolation if essential self-intersection are absent.

### Treating alignments with gaps

Optimal structural superposition of two protein chains includes gaps in the alignments of the chains to compensate for the naturally occurring insertions and deletions in homologous protein chains. A canonical example is that a loop may be longer in one structure than in the other; but such that the long loop can be contracted into the shorter loop without causing self-intersections of the entire structure. A sequence or structural alignment determines a paring of only a part of the residues of the two proteins. We choose to fill out the alignment gaps using linear interpolation as explained by the following example involving a 12 residue long Chain 0 and a 10 residue long Chain 1 using the notation of the program TM-align:

**Table:** 

Backbone 0	1	2	3	4	5	6	7	8	9	–	–	–	10	11	12
TM align			.	:		:	:						:	:	
Backbone 1			1	2	–	3	4	–	–	5	6	7	8	9	10

The first aligned residue pairs between Chain 0 and Chain 1 are (3, 1), (4, 2) and (6, 3). We treat weakly aligned pairs, indicated with “.” by TM-align, as aligned pairs indicated by “:”. Residue 5 of Chain 0 is not aligned and we choose to align it with the midpoint $P^1_{2.5}$ between Chain 1’s second and third residue. The next two aligned pairs (7, 4) and (10, 8) leave 3 intervals open on Chain 0 and 4 intervals on Chain 1. By linear interpolation, Curve 0 needs to be traversed at 3/4 of the speed Curve 1 is traversed resulting in the re-parameterizations:

**Table:** 

New param.	1	2	3	4	5	6	7	8	9	10
Re-param. 0	3	4	5	6	7	7.75	8.5	9.25	10	11
IsAligned	1	1	0	1	1	0	0	0	1	1
Re-param. 1	1	2	2.5	3	4	5	6	7	8	9

The re-parameterized Curve 1 is the piecewise linear curve connecting the points:

**Table:** 

${\tilde{P}}^1_1=P^1_1$	${\tilde{P}}^1_2=P^1_2$	${\tilde{P}}^1_3=P^1_{2.5}$	${\tilde{P}}^1_4=P^1_3$	$\dots$	${\tilde{P}}^1_{10}=P^1_9$

The re-parameterized Curve 0 connects the points:

**Table:** 

${\tilde{P}}^0_1=P^0_3$	${\tilde{P}}^0_2=P^0_4$	${\tilde{P}}^0_3=P^0_5$	${\tilde{P}}^0_4=P^0_6$	${\tilde{P}}^0_5=P^0_{7}$
${\tilde{P}}^0_6=P^0_{7.75}$	${\tilde{P}}^0_{7}=P^0_{8.5}$	${\tilde{P}}^0_{8}=P^0_{9.25}$	${\tilde{P}}^0_{9}=P^0_{10}$	${\tilde{P}}^0_{10}=P^0_{11}$

The piecewise linear curve connecting the ${\tilde{P}}_i^0$’s differs slightly form the original curve as e.g. a corner around $P_8^0$ is cut when connecting ${\tilde{P}}^0_6=P^0_{7.75}$ to ${\tilde{P}}^0_{7}=P^0_{8.5}$. However, it is trivial to morph the one curve into the other and the thickness of the protein backbone guaranties that no self-intersections can occur when doing this. The structural alignment thus gives rise to studying the self-intersections of the linear interpolation

$$\begin{aligned} P(t,a)=(1-t)P^0_{{\text {RePar}}^0(a)}+tP^1_{{\text {RePar}}^1(a)}, \end{aligned}$$

for $t\in [0,1]$ and $a\in [1;m]$, where *m* is determined by the alignment On each line segment the function IsAligned(a) is given by linear interpolation of the values zero (for not aligned) and one (for aligned) at the endpoints of the line segments. In particular, it is one on line segments connecting aligned residues and zero when connecting non-aligned residues.

A self-intersection for parameter values $(a_k,b_k)$ gives the sum ${\text {AlignedSum}}(k)={\text {IsAligned}}(a_k)+{\text {IsAligned}}(b_k)$. We call the self-intersection aligned-aligned if ${\text{AlignedSum}}(k)\; \ge \;1.5$, aligned-gap if $1.5> {\text {AlignedSum}}(k) > 0.5$ and gap-gap if ${\text{AlignedSum}}(k)\; \le \;0.5$. The minimal allowed distance, $d_{min}$, between points along the backbone curve used to define the overlap are given by linear interpolation of the discrete $d_{min}$ values after the re-parametrization. E.g., $d_{min}$ for two points 4.80 line segments apart along the backbone curve is set to $3.51{\AA }$ found by interpolating $d_{min}= 3.47{\AA }$ and $3.52{\AA }$ for points being 4 and 5 line segments apart respectively.

A loop-contraction combined with other deformation between two structures likely lead to a pair of self-intersections of type $\Omega _2$. If just one of these self-intersections involves the aligned parts and the other not, then the aligned-aligned self-intersection is probably essential when only considering aligned parts of the morph whereas it is removable if all self-intersections are considered. Beside the goal to tell if alignment gaps are topologically similar, this is a reason not to restrict the morph to the aligned parts.

### Applying curve smoothening

We represent each protein structure by either the positions of its alpha carbon atoms $C_i^\alpha$ or by a smoothened curve. In the smoothening all alpha carbons, except for the first two and the last two are replaced by the fixed convex combination $P_i=\frac{C_{i-2}^\alpha +a C_{i-1}^\alpha +b C_{i}^\alpha +a C_{i+1}^\alpha +C_{i+2}^\alpha }{2+2a+b}$. The constants $a=2.4$ and $b=2.1$ are chosen to minimize the total curvature of a collection of protein structures [18]. All linear interpolations from an alpha carbon curve to its smoothened curve are self-intersection free for the protein structures used here. The smoothening straightens both alpha helices and beta-strands, see Fig. 6. The smoothened representation resembles typical cartoon pictures of protein structures and has the advantage often to make it possible to follow an interpolation visually, see Fig. 6. Furthermore, it results in fewer interpolation self-intersections. For the smoothened representation, the overlap is based on the minimal distances 1.0, 2.1, 3.0, 3.4, 3.6Å for residues with indices $|i-j|=1,\dots ,5$ apart and 3.7Å otherwise. A self-intersection check may be avoided if the line-segments are shorter than 3.5Å and the sum of the endpoints overlap is $<2.1$Å.

## Results

When performing linear interpolation between more and more distant structures, overlap will start to grow and eventually self-intersections that can be removed using $\Omega _1$ and $\Omega _2$ moves will emerge. At first, each $\Omega$ move involves only a small part of the backbone. For a self-intersection with data $(a_k;b_k; sign_k; t_k )$ we defined the backbone length of the corresponding $\Omega _1$ move as $b_k-a_k$ and for an $\Omega _2$ move it’s backbone length is also the (real) number of line segments it involves. When interpolating structures that are more distant these backbone lengths may grow and eventually obstructions to the $\Omega _1$ and $\Omega _2$ moves may occur. This we now illustrate with a few examples with high sequence identity. The $^*$-marked examples have 100% sequence identity and are taken from the Sequence-Similar, Structure-Dissimilar Protein Pairs in the PDB found by Kosloff and Kolodny [2].

### Examples with high sequence identity

The A and B chains of the open and closed forms of adenylate kinase, 4AKE and 1ANK, both have RMSD$\approx 7\AA$. The interpolation cause no overlap of the alpha carbon curves, and next to no mean overlap of $10^{-3}\AA$ for the smooth curves and consequently self-intersection checks are not needed. We may conclude that both the A and B chain interpolations are easy to perform. We expect the same conclusion for a hinge-motion of relatively ridged sub-domains since neighboring residues perform almost identical motions. The $5.3\AA$ RMSD motion between the glutamine binding protein’s structures 1GGG and 1WDN with and without ligand is an example of this. The RMSD$\,=\,8\AA$ interpolation of hepatitis C virus between the A chain configurations 1A1Q and 1$\hbox {JXP}^*$ has a mainly local overlap of mean $0.11\AA$ and only 5 self-intersection checks are needed for the alpha carbon curve interpolation. The alpha carbon curve interpolation is self-avoiding. The large, RMSD$\,=\,23\AA$ motion between the (un-) and phosphorylated forms of $\hbox {Odhl}^*$ is shown in Fig. 7 left. The free arm performs a long motion and the remaining compact domain rotates close to a third of a full rotation in the RMSD superposition. The linear interpolation of this large rotation contracts the structure and causes $1.0\AA$ mean overlap ($0.6\AA$ for the smooth curve). Only the interpolation of the alpha carbon curves has self-intersections. Its 3 self-intersections may be removed by one $\Omega _1$ and one $\Omega _2$, move each of backbone length less than 4 line segments. Note, that all interpolations considered so far are ‘easy to perform’ even if some of them involve larger motions.

The ${\text {RMSD}}=14\AA$ linear interpolation between two configurations of the 60 residue SH3 domain is shown in the middle of Fig. 7. The alpha carbon and smooth backbone interpolations both have one self-intersection, that in both cases may be resolved by an $\Omega _1$ move involving about 15 residues or by an end-contraction of backbone length counted as twice the 10 residues it involves. Hence, this self-intersection is considered as non-essential if ${\text {MaxLength}}$ e.g. is 16 which may be larger than some users will allow. Finally the ${\text {RMSD}}=13\AA$ interpolation from the folded 1D2Z C chain to the 52 residue long helix 1IK7 A $\hbox {chain}^*$ is shown to the right in Fig. 7. The folded structure is monotone in the direction of the helix and consequently no self-intersections occur even though most consider the topology changed.

TM-align seldom aligns the entire shorter chain and introduces generally more gaps than the global RMSD alignment. The resulting interpolations therefore are different from the RMSD based above. TM-align aligns 181/183 of the 214 residues of the A/B chains of 4AKE and 1ANK and the re-parameterized curve has 220/220 vertices covering the entire original chains. For the alpha carbon curves the gaps cause mainly local overlap of mean $0.79/0.74\AA$ and one $\Omega _1$ removable self-intersection. The same is found in the A chain interpolation between 1A1Q and 1$\hbox {JXP}^*$ and between 1GGG’a A chain and 1WDN, where apart from the local overlap of the alpha carbon interpolation, both the alpha carbon and smooth curve interpolations have non-local overlap. The situation is quite different in the case of Odhl where TM-align aligns only the compact and almost unchanged part of the structure. For the SH3 domain, 35 of the first 36 residues are aligned by TM-align together with the weakly aligned 47’th residue. The one self-intersection is again present; this time in the alignment gap and its presence is thus due to the weak alignment of residue 47. In the 1IK7-1D2Z C chain example TM-align aligns only 18 almost consecutive residues identifying two helical segments. These examples fall in two main cases. In the first, the TM-alignment covers most of the aligned chains. Here the gaps in the TM-alignment, that are not taken into account in the TM-superposition, cause more overlap especially for the alpha carbon curve interpolations of which some have a local $\Omega _1$ removable self-intersection. The other main case is when TM-align identifies a structurally conserved region whose interpolation is trivial but where the global RMSD alignment shows that the full structures are connected by relatively long but obstruction free interpolations.

### Detecting knottedness

The D chain of 2FG6 contains a right handed trefoil knot located in the interval from the 171th to the 238th residue [26]. We align the 83 residues 162 to 244 of 2FG6’s D chain that contain the knot to 408 sequence class representatives of CATH 2.4 domains of compatible lengths from 75 to 100 residues. Using global RMSD superposition, all these interpolations have at least 1 self-intersection with and average of 8.4 intersections (3.6 for the smooth representation). In the following *n*(*m*) is short hand for *n* for the alpha carbon curve and *m* for the smooth representation. Depending on ${\text {MaxLength}}$ almost all morphs have at least one essential self-intersection. E.g. for ${\text {MaxLength}}=10$ only 3(1) domains of topologies 3.40.30 and 3.66.20 are morphed into the trefoil knot without causing essential self-intersections. For ${\text {MaxLength}}=20$ there are 11(11) such cases. We have cut 2FG6’s D chain relatively close to its trefoil knot, and it may become unknotted by a similar size end-contraction. This effect is very pronounced as self-avoiding morphs are found in 6(5), 34(25), 109(81), 177(151), 325(318) of the 408 cases for ${\text {MaxLength}}= 5, 10, 14, 16, 20$ respectively when end-contractions up to backbone-length ${\text {MaxLength}}/2$ are allowed. Without end-contractions, a self-avoiding morph is found only in cases that firstly, are unknotted by the original interpolation by moving an end through an enclosing loop, and that secondly also have a sufficiently large ${\text {MaxLength}}$ to avoid other self-intersections locally. For ${\text {MaxLength}}=20$ and with end-contraction of at most 10 residues, the end-contraction may unknot the knot and hereafter the 20 residue long Reidemeister moves are powerful enough to find a self-avoiding morph to most of the other folds. Hence, to capture the characteristics or topology of a fold; one has to set the maximal allowed end-contraction in concordance with how tightly the representative domain is cut. Similarly, many TM-alignment windows are too short to contain the knot, in which case fewer morph obstructions are found.

### Data

We pick a representative set of protein structures by taking the sequence family representative CATH2.4 domains [37] restricting to cases where it is gap free and has between 75 and 150 residues. These are clustered at 60% sequence homology and give 1034 domains representing 1034 sequence families, 521 homology families (H-classes), 281 topologies T-classes, 23 architectures and the 4 main classes. If the longest of a pair of domains is at most 10% longer than the shorter domain, we find the alignment of the entire shorter domain with minimal RMSD allowing one inner gap in this alignment. The choice of limited differences in domain lengths together with shorter but frequent domains is done to make global alignment in the best possible agreement with the structural classification. The global RMSD alignment use exhaustive search and is time consuming. Hence, the choice of domain pairs is also necessary to make the global alignment practical. For each of these 142068 alignments we calculate the RMSD together with overlap and topological obstructions found in the corresponding linear interpolations. We do the same based on the TM-alignments using its standard parameters. We find 135698 inter T-class pairs, 6370 intra T-class pairs, and 1304 intra H-class pairs giving 5066 intra T-class pairs that are not inter H-class pairs. All data from these alignments are provided as Additional files 1, 2, 3, 4 that generate the figures on CATH alignments below.

### On inter and intra fold class morphs

This section concerns the 142,068 structural alignments of CATH domain pairs of similar chain length. For the alpha carbon curves 29% of the total overlap is local caused by residues with indices $|i-j|\le 4$. Relatively large local metric changes when alpha helices are involved is the main contributor to the local overlap. For comparison, only 7% of the total overlap is local for the smooth curves. The non-local contributions coming from aligned regions are highly correlated (0.97) between the alpha carbon and smoothened curves.

#### Self-intersections and alignment gaps

A structure with $n+1$ alpha carbons has *n* line segments and $n(n-1)/2$ line segment pairs. If a self-intersection has ${\text {AlignedSum}} \ge 1.5$ then we say it is between two aligned parts of the structure. Otherwise, a least one alignment gap is involved. Our global RMSD alignment allows one gap that usually is too short to make intersections with itself in the linear interpolation. One thousand aligned line segment pairs on average cause 2.2 self-intersections and gap-involving line-segment pairs cause 1.6. The smooth representation generally cause fewer self-intersections and the corresponding numbers are 0.81 and 0.52 respectively. When using TM-align we connect all inner gaps in the alignments to check if they cause topological changes. We use the Aligned Window Fraction (AWF) given by the shorter aligned window length divided by the shorter chain length to characterize the coverage of the aligned windows. On average 55% of the residues in the shorter chain are aligned and AWF is 0.8 on average. From allowing similar size end-contractions on the knotted example above, one hereby expects and finds significantly fewer self-intersections. One thousand aligned line segment pairs on average cause 0.17 (0.048 smooth) self-intersection where gap-involving line-segment pairs cause 0.73 (0.22 smooth). TM-align aligns pairs of alpha carbons that in the final superposition are at distance at most 5Å. This short distance does not prevent self-intersections when interpolating the superimposed structures. In 142068 TM-aligned domain pairs, we find 42165 (12103 smooth) self-intersections of aligned parts. For both alignment methods, the alpha carbon curves cause almost 3 times as many self-intersections as the smooth curves.

#### Essential self-intersections

Figure 8a shows the average number of essential self-intersections as function of the maximal allowed backbone length, ${\text {MaxLength}}$, of $\Omega _1$ and $\Omega _2$ moves. The original number of self-intersection is found for ${\text {MaxLength}}=0$. There are clearly more self-intersections for the alpha carbon representation, e.g. on average 13 between fold classes compared to the 4.9 for the smooth representation, and there is a relatively low correlation of 0.68 between the numbers of self-intersections in the two representations. However, the essential self-intersection numbers of the two representations are closely related for ${\text {MaxLength}}$ at least four. See Fig. 8a, b. Their correlation is 0.92 for $4\le {\text {MaxLength}}\le 6$ and drops slowly down to 0.79 for ${\text {MaxLength}}=20$.

The shorter TM-alignments give fewer essential self-intersections than the global RMSD alignments as expected. For the TM-alignments ${\text {MaxLength}}$ needs to be larger to remove the additional self-intersections of the alpha carbon curves. See Fig. 8d, e. For both alignment methods, an essential self-intersection for smooth curves generally implies one for the alpha carbon curves and the notion of essential self-intersections therefore seems robust when restricted to one alignment method. Both the alignments and superpositions provided by the two alignment methods may be very different. One should thus not expect essential self-intersections to agree between them. However, if there is an essential self-intersection in the TM-aligned window, then it is more likely than average to find one in the RMSD alignment, Fig. 8b. If the TM-aligned window covers most of the structure also the opposite tendency is found, Fig. 8e.

Figure 8c shows the average number of essential self-intersections as function of the aligned window length and Fig. 8f shows the ratio between them. TM-align can avoid self-intersections by choosing a shorter aligned window. But also in cases where the TM-alignment window is almost global, it results in fewer essential self-intersection. See Fig. 8f. Possibly both TM-aligns freedom to optimize the alignment using multiple gaps and its choice of superposition result in fewer essential self-intersections. Figure 8f shows that for larger values of ${\text {MaxLength}}$ the local Reidemeister moves manage to compensate for many of these additional self-intersections caused by the lack of gaps in the global alignments.

#### Morphability and fold classification

Interpolations between pairs of homological structures have essential self-intersections relatively often. Figure 6 shows an example where the sole self-intersection may be removed by an $\Omega _1$ move involving half of the chain or by a relatively large movement of one of the long ends. This author finds it debatable if these two domains should share fold class. For ${\text {MaxLength}}=10$ as many as 13% of all H-pair morphs have essential self-intersections, but usually for greater RMSD than this example as seen from Fig. 9. The fraction of H-pair morphs with essential self-intersections varies between the classes. E.g., 5 out of the 14 alignments in class 1.20.120.200 self-intersects as seen on Fig. 6. In total 47 of the 512 H-classes with intra H-class morphs have essential self-intersections for ${\text {MaxLength}}=10$, but most classes require more data to be studied in detail. See the included supplementary data. Due to the large global RMSD values of interpolations with essential self-intersections, TM-align typically aligns too few residues to capture the eventual topological dissimilarity. The most striking finding is not that more than half of all intra T-class global RMSD morphs have essential self-intersections, but that many inter T-pair morphs do not. For smooth representation and ${\text {MaxLength}}=10$, 868 of 879 H-pairs, 1189 of 1295 non-H but T-pairs and 1831 of 3852 inter T-pairs with RMSD $\le 10$ do not cause essential self-intersections. Hence, under this RMSD restriction, most intra T-pairs, in total 2057 of 2174, are morphable, but there is a compatible number of self-avoiding morphs of similar length into other fold classes emphasizing the continuous nature of protein folds. An example is the 7.1Å global RMSD interpolation between the 6 helix domain 2ygsA0 and the 2 helix and 4 beta-strand domain 1ha101 which is self-intersection free both in the alpha carbon and smooth representation. The 0.49Å(0.03Å smooth curve) mean overlap has very little 0.03Å(0.001Å) non-local contribution. Hence, even if their local geometry and hydrogen bonding patterns are very different, their full backbones are of typical H-pair distance in our data set, and the linear interpolation causes only minor local steric problems.

#### Morph lengths

In case no essential self-intersection is found, the original $L^1$ morph length plus the price of the $\Omega _1$ and $\Omega _2$ moves is an estimate of the length of a self-avoiding morph for the piecewise linear backbone curve not taking into account the thickness of a protein chain. The shortest overlap free distance is $d=3.7\AA$. Hence, each point on the back bonecurve has to traverse half the circumference of a circle with radius *d*/2 to go from over sliding to under sliding and staying at an overlap free distance. One may therefore add the penalty $\pi *d/2*|b_k-a_k+1|$ for $\Omega _1$ moves and $\pi *d/2*|b_j-a_j+b_k-a_k+2|$ for $\Omega _2$ moves. The total overlap is the sum of the distances each pair of amino acids need to get further apart during the morph in order to get an overlap free morph. One may therefore wish to add and possibly weight the ${\text {AverageOverlap}}$ to the morph-length estimate. Similarly, one may introduce a weight for the end-contraction length and for the penalty for net torsional effects. Figure 9 shows the average contribution from one residue to morph lengths on intra T-morphs as function of ${\text {MaxLength}}$ for the smoothened backbone curves. Clearly shorter morphs are found for larger ${\text {MaxLength}}$ as end-contractions are longer than local Reidemeister moves. Inter T-class morphs are on average $26\AA$ for ${\text {MaxLength}}=3$ and $21\AA$ for ${\text {MaxLength}}=20$, that is only slightly longer than the intra T-class morphs. The global RMSD alignment thus do not find a drastic difference in the lengths of inter and intra fold class self-avoiding morphs. TM aligns approximately half of the residues, namely those superimposed within $5\AA$ distance. The morphs lengths of the aligned windows are therefore considerably shorter than those obtained using the global RMSD alignment. The TM-alignment based morph lengths increase mainly due to the gaps in the alignment and to the topological obstructions of the morph. For ${\text {MaxLength}}=3$ the average morph lengths are 3.2, 6.4 and 13.2$\AA$ per residue for intra H, inter H but intra T, and inter T class morphs respectively. For ${\text {MaxLength}}=20$ the similar numbers drop to 3.0, 5.5 and 11.0$\AA$.

## Discussion

We are handling a paradox by trying to establish a “topological” distinction in the path connected space of protein structures, and we emphasize that the set of essential self-intersections we find are only local obstructions in the non-convex but connected space of protein embeddings. If a current alignment score function shows high similarity then the two configurations are close, at least, in the space of immersions, which allows self-intersections. We offer to test if the linear interpolation between the two structures is self-avoiding and if not then we offer an algorithmic search for alternative morphs through embeddings in a neighborhood of the linear interpolation. We divide the self-intersections into two sets. The set of non-essential self-intersections contains the maximal number of self-intersections that can be avoided using a sequence of $\Omega _1$ and $\Omega _2$ moves. The remaining self-intersections are called essential. There are three possible reasons why a given self-intersection is marked essential. The first is that a needed $\Omega$ move involves larger parts of the protein chain than permitted by the user. The second is that there is a “topological” obstruction to a needed $\Omega$ move. The third is that the self-intersection can be avoided but it is cheaper to avoid other self-intersections. The ”topological” obstruction is a case where the constructed $\Omega$ move will cause at least one new self-intersection and is technically found as an intersection between a constructed disk-surface and the protein structure. However considering protein structures as open curves any such intersection can be avoided by deforming the disk-surface. In most cases these intersections need to be pushed past at least one chain terminal, and this results in a large move that is more efficiently resolved by an end-contraction. In some cases two backbone-surface intersections lie close along the backbone and a smaller alteration of the surface may avoid them both - think of untying a slip-knot. In this study, there has most often been only one backbone-surface intersection. Hence, its a relatively rare situation that the obstructions to an $\Omega$ move can be avoided locally and this lies outside the scope of this work, but it may become more important if longer ${\text {MaxLength}}$ are wanted. Algorithmically we have decided to keep our untying moves local along the backbone to ensure they can be treated in one-pass. Mathematically, our novel notion of essential self-intersection is hereby not a topological but a geometric notion saying that any self-avoiding morph between the two configurations has to go further than a given threshold away from the linear morph path. It is our hope and main aim that this tunable threshold reflects a general perception of when two structures have different folding paths.

In the example shown on Fig. 11csgA0 is resolved by x-ray diffraction and 1jli00 by NMR. From the alignment its clear that the two structures have similar distance matrices and residue contacts leaving the possibility that the morph self-intersection points to a modeling error in one of the two structures. The smoothened representation makes visual inspection of morphs significantly easier. The speedup of calculations due to fewer self-intersections in this representation was however neutralized as the filtering of potential self-intersections by overlap is less efficient. The overlap is the sum of the steric clashes during an imagined linear interpolation between two aligned and superimposed structures. It is important algorithmically and may be interesting structural biologically. Even in a case where the backbone curve morph is self-avoiding, the overlap holds information in addition to the morph length. We saw examples of long overlap free morphs between sequence identical highly flexible structures. In these morphs, a neighborhood of most residues undergo a motion close to a rigid motion. This cause both little overlap and that the shorter internal distances are subject to only smaller changes. Hence, a structural comparison based on local internal distances as FlexE will also detect that neighboring residues perform similar motions. Thus, the combined information of RMSD and overlap may be similar that of RMSD and FlexE in such cases.

## Conclusion and future work

We present a number of measures that quantify obstacles to deform a protein structure to an aligned and superimposed structure and show they give significant additional information to the distances usually used in protein structure alignment score functions. We study the linear interpolation between two superimposed structures. First, we quantify the steric problems of this linear interpolation by finding the shortest distance between each amino acid pair during the interpolation and compare it to distance constraints found in native protein structures. Larger deformations between sequence similar native structures cause little overlap and are recognized as longer but simple morphs. Next, we find all self-intersections of the protein backbone during linear interpolation between two aligned structures. We introduce 3-dimensional versions of Reidemeister moves each capable of avoiding one or two self-intersections by altering the original linear morph. Near a protein terminal an end-contraction can also be used to avoid a self-intersection. We calculate the length of each of these morph alterations and solve the optimization problem to avoid the maximal number of self-intersections at the lowest additional morph length. If this results in a self-avoiding morph the structural similarity found by the alignment program is verified for the entire aligned windows including all inner alignment gaps. Otherwise we find a smallest set of essential self-intersections that cannot be avoided. We demonstrate that this novel notion of essential self-intersections is relatively robust, e.g., under a change in the representation of protein chains. The user may input the maximal allowed chain length of Reidemeister moves and of end-contractions. We hope this allows users to tailor an appropriate notion of when two structures have different threading for the application at hand.

We find as expected that morphs from unknotted proteins to a given knotted protein have essential self-intersections except for a few cases where the morphs actually untie the knot. Using a global RMSD alignment and a set of sequence representative CATH domains, we find a significant fraction of all homologous protein structure pairs separated by at least one essential self-intersection and give examples of homologous pairs that most will considered as different threaded despite having lower RMSD. Our perhaps most interesting finding is that many representatives of distinct folds may be morphed into each other using only smaller alterations of the direct linear interpolation. This supports the continuous view on parts of protein fold space.

The residues that are aligned by TM-align are superimposed within $5\AA$. We find 42165 (12103 smooth curve) self-intersections between aligned parts in 142068 TM-alignments. Thus this close distance still allows topological dissimilarity of the aligned parts. In the TM-alignments performed in this work approximately half of the residues are aligned on average. The TM-alignments result in fewer topological obstructions than expected alone from their shorter alignment length. An interesting subject for future research is therefor to develop methods that combine the superior structural alignment search of e.g. TM-align with the restriction to local topological similarity defined by the absence of essential morph self-intersections. The current algorithm is in Matlab and requires two superimposed structures and the alignment in TM-align format as input. It is available on request to the author. When a matching alignment method is developed, the software can stand-alone and will be release.

With fixed sequence alignment, protein structure prediction is a natural application of our present method as structural comparison is done for the entire structure. Capable of detecting differences in threading and pointing out where in the structure these problems occur, we expect our method to give an interesting supplement to the distance measures used to assess protein models as e.g. done in the Critical Assessment of protein Structure Prediction (CASP) experiments.

When combined with TM-align, our method can detect cases where structurally similar proteins have different folding pathways, which is important for understanding protein folding. In addition, alternate threading of a similar core structure may change dynamical and thereby functional properties. Further, we hope our focus on self-avoiding morphing may contribute to the development of alignment methods and to a more detailed picture of sequence to structure relationship for proteins and RNA.

## Supplementary information

**Additional file 1.** SupplementaryDataCATH2_4Domains.m. Matlab script that loads and explains all the raw data from all CATH2.4 alignments. It produces figures and may be altered to investigate the data further.

**Additional file 2. **Data from aligning CATH2.4 domains. NeamTM3to20NoEndContraction.mat Data from global RMSD and TM-align alignments of alpha carbon backbone curves. The maximal length of Reidemeister moves is varied from 3 to 20 and end-contractions are not allowed. Similar data for the smooth representation of the backbone curve are contained in NeamTMSmooth3to20NoEndContraction.mat and in NeamTMSmooth3to20EndContraction.matwhen allowing end-contractions of MaxLength=2/residues.

**Additional file 3. **Data from aligning CATH2.4 domains. NeamTM3to20NoEndContraction.mat Data from global RMSD and TM-align alignments of alpha carbon backbone curves. The maximal length of Reidemeister moves is varied from 3 to 20 and end-contractions are not allowed. Similar data for the smooth representation of the backbone curve are contained in NeamTMSmooth3to20NoEndContraction.mat and in NeamTMSmooth3to20EndContraction.matwhen allowing end-contractions of MaxLength=2/residues.

**Additional file 4. **Data from aligning CATH2.4 domains. NeamTM3to20NoEndContraction.mat Data from global RMSD and TM-align alignments of alpha carbon backbone curves. The maximal length of Reidemeister moves is varied from 3 to 20 and end-contractions are not allowed. Similar data for the smooth representation of the backbone curve are contained in NeamTMSmooth3to20NoEndContraction.mat and in NeamTMSmooth3to20EndContraction.matwhen allowing end-contractions of MaxLength = 2/residues.

## Data Availability

All data on the alignments of CATH2.4 domains are supplied as supporting material together with a Matlab-script for reading and handling the data.
